# Bio-inspired synthesis of palladium nanoparticles fabricated magnetic Fe_3_O_4_ nanocomposite over *Fritillaria imperialis *flower extract as an efficient recyclable catalyst for the reduction of nitroarenes

**DOI:** 10.1038/s41598-021-83854-1

**Published:** 2021-02-25

**Authors:** Hojat Veisi, Bikash Karmakar, Taiebeh Tamoradi, Reza Tayebee, Sami Sajjadifar, Shahram Lotfi, Behrooz Maleki, Saba Hemmati

**Affiliations:** 1 Department of Chemistry, Payame Noor University, Tehran, Iran; 2 Department of Chemistry, Gobardanga Hindu College, North 24, Parganas, India; 3 Department of Chemistry, Hakim Sabzevari University, 96179‐76487 Sabzevar, Iran

**Keywords:** Chemistry, Materials science

## Abstract

This current research is based on a bio-inspired procedure for the synthesis of biomolecule functionalized hybrid magnetic nanocomposite with the Fe_3_O_4_ NPs at core and Pd NPs at outer shell. The central idea was the initial modification of magnetic NP by the phytochemicals from *Fritillaria imperialis *flower extract, which was further exploited in the green reduction of Pd^2+^ ions into Pd NPs, in situ. The flower extract also acted as a capping agent for the obtained Pd/Fe_3_O_4_ composite without the need of additional toxic reagents. The as-synthesized Fe_3_O_4_@*Fritillaria*/Pd nanocomposite was methodically characterized over different physicochemical measures like FT-IR, ICP-AES, FESEM, EDX, TEM, XPS and VSM analysis. Thereafter, its catalytic potential was evaluated in the reduction of various nitrobenzenes to arylamines applying hydrazine hydrate as reductant in ethanol/water (1:2) medium under mild conditions. Furthermore, the nanocatalyst was retrieved using a bar magnet and recycled several times without considerable leaching or loss of activity. This green, bio-inspired ligand-free protocol has remarkable advantages like environmental friendliness, high yields, easy workup and reusability of the catalyst.

## Introduction

**Figure 1:**
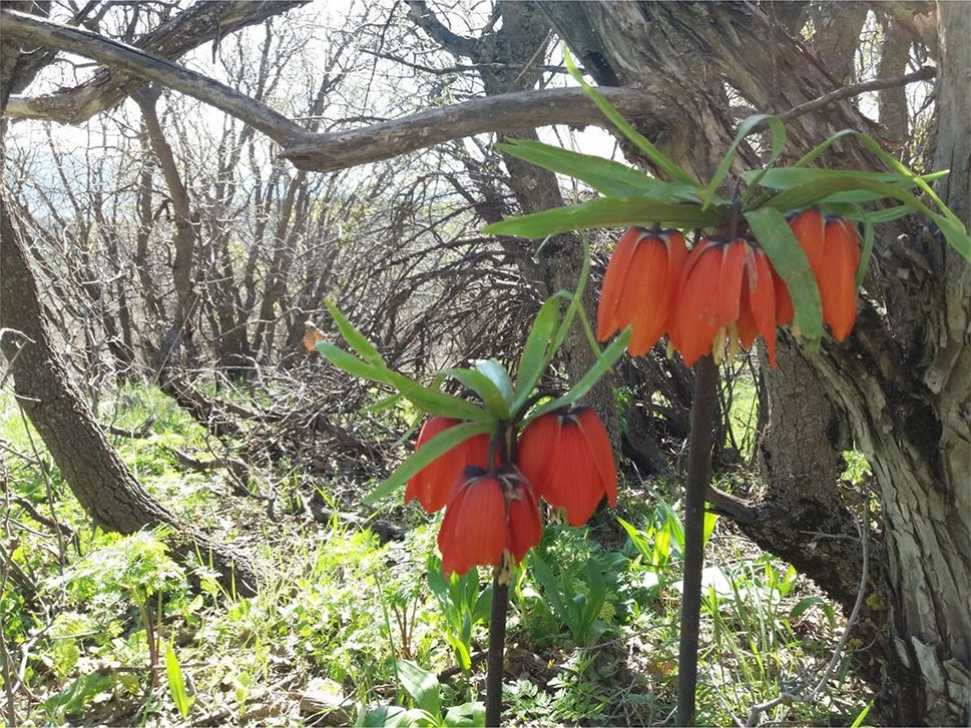
* Fritillaria imperialis* flower image.

The catalytic society in recent days has shown significant interest and applied extensive thrust on the development of engineered heterogeneous catalysts as compared to their homogeneous analog. Their easy handling and facile isolation from the reaction mixture has made them advantageous^1–4^. Among them, the magnetite nanoparticles (MNPs) have acquired remarkable attention as catalyst support or as the core of nanohybrid composites to serve as a potential reusable green catalyst^5–7^. The features like easy availability, abundance, small size thereby high surface area, excellent reactivity, high biocompatibility, good magnetic permeability, presence of plenteous hydroxyl groups for surface engineering and straightforward magnetic isolation has made them a fascinating material^8–14^. However, due to high surface energy, they are highly prone to self-aggregation which reduces their catalytic activity significantly^15–18^. This is somewhat minimized by surface functionalization. In recent years, the biogenic green approach for synthesis of NPs has come into prominence^19–24^. Plants have been a ubiquitous and rich source in this regard. There are reports of using plant leaves, fruits, flowers, barks, and roots’ extract as the cheap and abundant precursors of corresponding biomolecules for functionalization^25–33^. Following the trend of our earlier report towards the bio-inspired synthesis of stable and active nanocomposite catalysts^34–40^, we demonstrate herein the *Fritillaria imperialis* flower as bio-resource to fabricate Fe_3_O_4_ NP. The flower is grown widely in the plateau areas of Turkey, Iraq and Iran border and Himalaya foothills (Fig. 1). The herb contains numerous phytochemicals including polyphenols, flavonoids, mild acids alkaloids and terpenoids. We further modified the biomolecule supported NPs by fabricating its exterior layer with tiny Pd NP as active catalytic phase. Finally, the catalytic application of the magnetic biogenic nanocomposite was demonstrated in the reduction of nitroarenes, a fundamental and significant chemical reaction in various organic transformations^41,42^. Particularly, 4-nitrophenol is a detrimental organo-pollutant in water and dreadful for all living creatures^43,44^. The reduced amines find wide applications in the synthesis of fine chemicals, agrochemicals, pharmaceuticals, dyes, polymers, pesticides, cosmetics and photography^45–48^. In view of such consequences we designed our catalyst to carry out the reduction in a facile and green chemical pathway using hydrazine hydrate (N_2_H_4_·H_2_O) as a mild and effective reductant. The magnetic core helps in efficient and effortless recoverability of the catalyst from the reaction mixture. Our protocol has been so proficient that a wide variety of nitro compounds has been converted to resultant amines within quick interval in aqueous ethanol producing outstanding yields and TOF.

## Experimental

### Synthesis of magnetite NPs

Following a typical co-precipitation method, a mixture of FeCl_2_·4H_2_O (2.0 g) and FeCl_3_·6H_2_O (5.2 g) were taken into deoxygenated water (25 mL) containing few drops of conc. HCl and subsequently, 250 mL of 1.5 M NaOH solution was added dropwise. The whole mixture was stirred vigorously at 60 °C. Immediately, brown colored Fe_3_O_4_ NPs were formed which were isolated using a magnetic stick. It was washed thrice with 200 mL deionized water and dried in air at 40 °C.

### Preparations of Fe_3_O_4_@***Fritillaria*** using the plant extract

0.5 gm *Fritillaria* flower powder was extracted into 50 mL of Milli-Q water by swirlling at 50 °C for 20 min. It was filtered over Whatman 1 paper and the filtrate was centrifuged at 4000 rpm for 5 min to precipitate out the impurities. The clear upper layer was preserved for the next step. For the preparation of Fe_3_O_4_@*Fritillaria* NPs, the magnetite NPs (0.5 g) were first dispersed in water by sonication for 20 min and the flower extract was added dropwise into it. The mixture was then stirred for 24 h at room temperature. Finally, the Fe_3_O_4_@*Fritillaria* nanocomposite was collected magnetically, washed thoroughly over DI-H_2_O and dried in vacuum at 40 °C overnight.

### Preparation of the Fe_3_O_4_@***Fritillaria***/Pd NPs

**Scheme 1:**
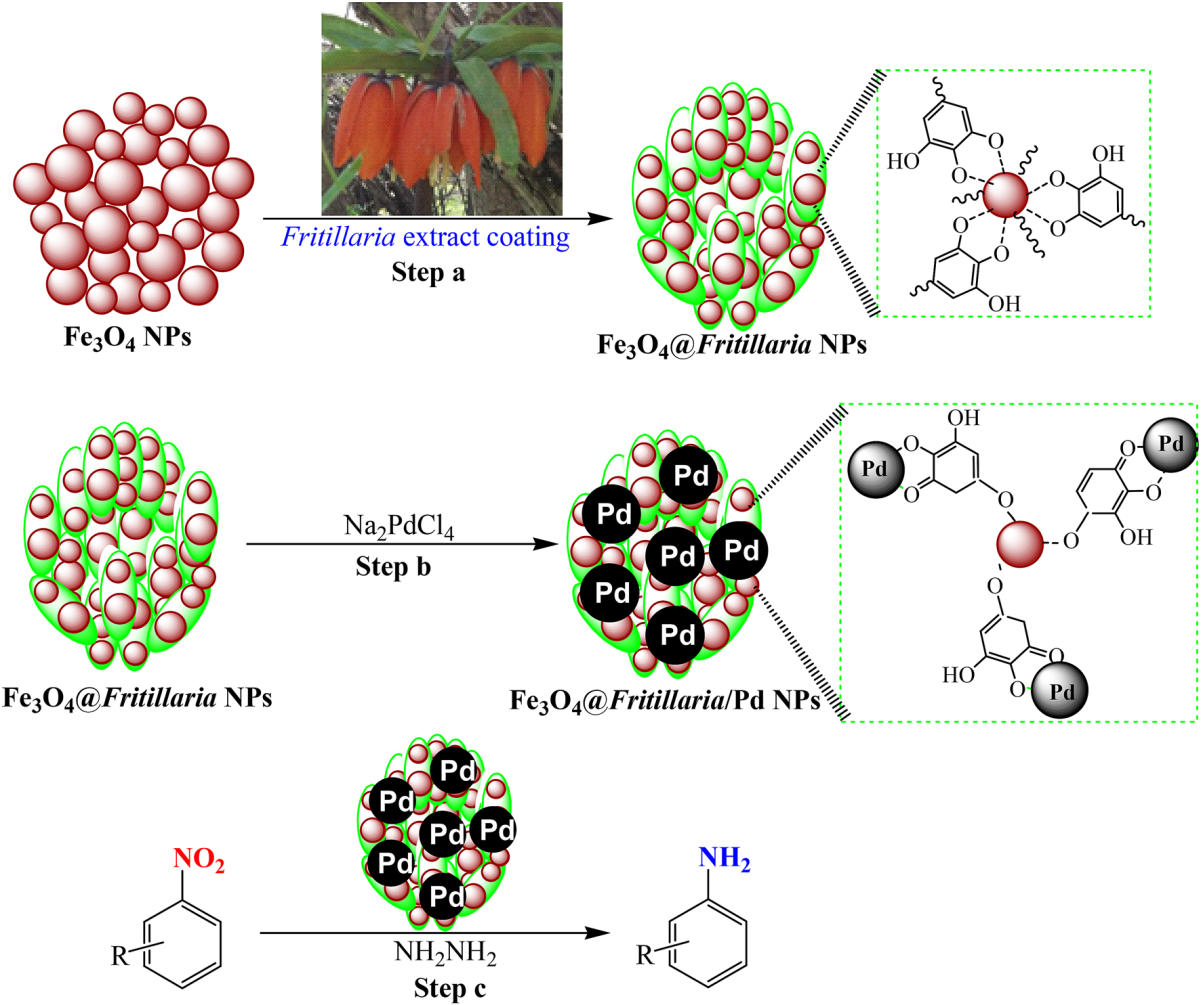
Schematic representation for the synthesis of catalyst and its application; step a: synthesis of Fe_3_O_4_@*Fritillaria,* step b: synthesis of Fe_3_O_4_@*Fritillaria/Pd*, step c: Reduction of nitroarenes over the catalyst.

Five gram of the Fe_3_O_4_@*Fritillaria* NPs was dispersed over deionized water (100 mL) in sonicator for 20 min. An aqueous solution of Na_2_PdCl_4_(40 mg in 20 mL H_2_O) was poured into dispersion and refluxed for 12 h to assure the complete reduction of Pd(II) ions. The Fe_3_O_4_@*Fritillaria*/Pd nanocomposite was isolated as previous, rinsed with H_2_O/acetone mixture to eliminate the adhered organic substances and dried likewise. The whole preparative schedule has been presented in Scheme 1. Pd content in the material was 0.08 mmol/g, analyzed by ICP-AES analysis.

### Catalytic reduction of nitrobenzene

In a stirring solution of nitrobenzene (1 mmol) and Fe_3_O_4_@*Fritillaria*/Pd nanocomposite (0.1 mol% Pd, 13 mg) in H_2_O/EtOH (2:1, 3 mL), the reducing agent NH_2_ NH_2_. H_2_O (3 mmol) was slowly dropped and the mixture was refluxed at 80 °C. After completion (by TLC, n-hexane/EtOAc: 5/2), EtOAc was added to the reaction mixture and stirred well. After removing the catalyst over a magnet, the water in reaction filtrate was soaked over anhydrous Na_2_SO_4_. Finally, the collected organic layer was concentrated to have pure aniline in 96% yield.

## Results and discussion

### Catalyst characterizations and data analysis

The current work illustrates an environmental friendly and green protocol involving *Fritillaria* flower extract to fabricate the ferrite MNPs surface and further to introduce stable Pd NPs. The biomolecules of *Fritillaria* flower has a significant tendency to accumulate over Fe_3_O_4_ MNPs. The polyphenolic compounds of the flower extract contain hydroxyl and ketonic groups that chelate Pd^2^^+^ ions and subsequently reduce them green metrically (Scheme 1). The structural and physicochemical characteristics of the nanomaterial was characterized using diverse analytical techniques like FT-IR, ICP-AES, FE-SEM, TEM, EDX, XPS and VSM studies.

**Figure 2:**
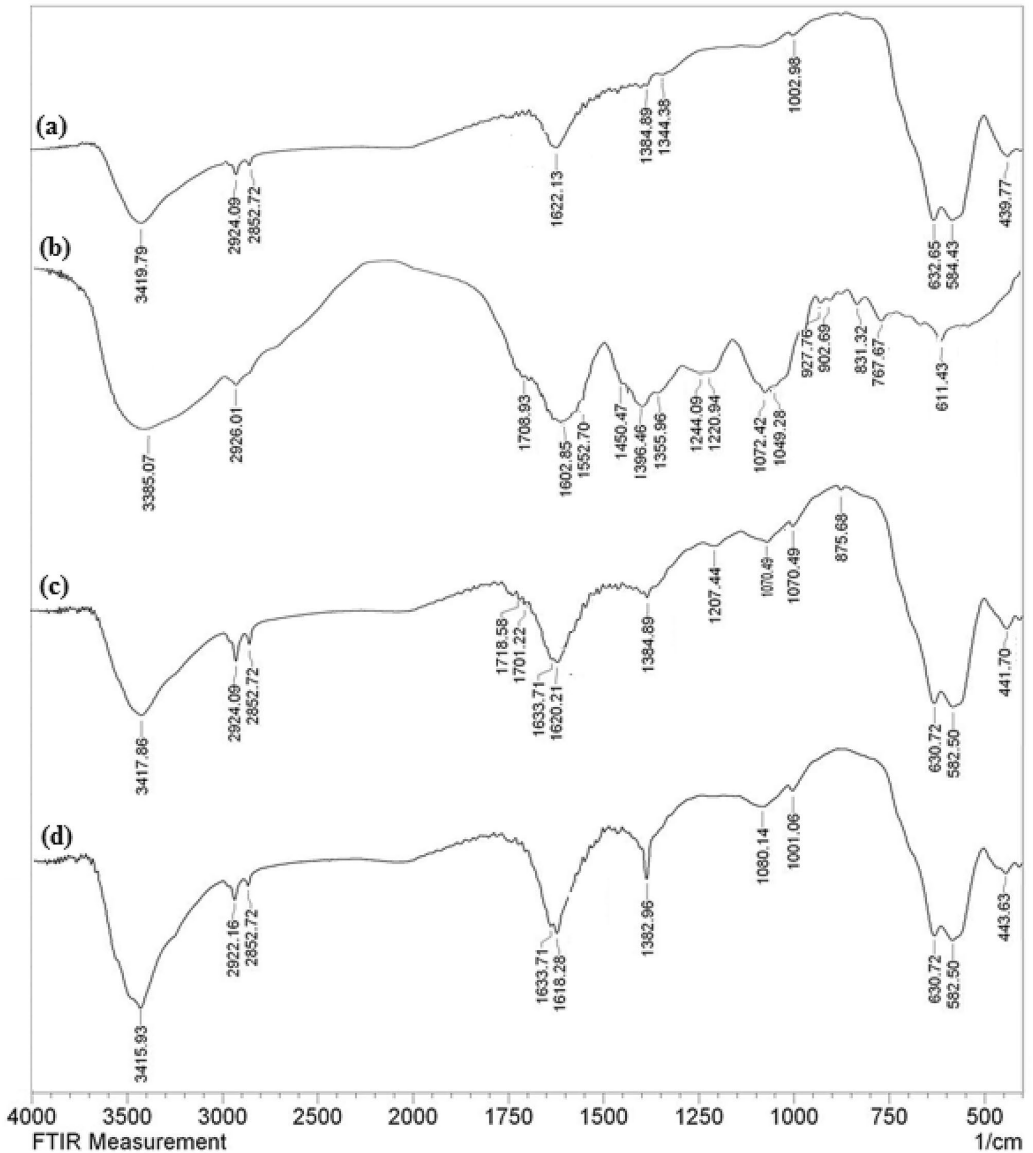
FT-IR spectrum of (**a**) Fe_3_O_4_, (**b**) *Fritillaria* extract, (**c**) Fe_3_O_4_@*Fritillaria* NPs, and (**d**) Fe_3_O_4_@*Fritillaria*/Pd NPs.

Figure 2 depicts comparative FT-IR spectra of bare Fe_3_O_4_ NPs, *Fritillaria* extract, Fe_3_O_4_@*Fritillaria* and Fe_3_O_4_@*Fritillaria*/Pd nanocomposite in order to illustrate the stepwise synthesis. In the spectrum of Fe_3_O_4_ NP (Fig. 2a), two broad peaks at 1622 and 3419 cm^−1^ correspond to the physisorbed H_2_O and the surface OH groups. The characteristic peaks appeared at 584 and 439 cm^−1^ are due to the stretching and bending vibrations of Fe–O bond. Pure Fe_3_O_4_ structure is characterized by a peak at 632 cm^−1^. Figure 2b represents the spectrum of *Fritillaria* extract which displays the significant peaks at 3385 cm^−1^ due to O–H groups of polyols^49^ and C-H stretching vibration from hydrocarbons and flavonoids at 2926 cm^−1^^50^. Additionally, due to the presence of quinones, ketones, and carboxylic acids functions in the biomolecules contained in it, the distinctive peaks of C=O and O–C–O appears at 1709 cm^−1^ and 1072 cm^−1^ respectively^51^. An FT-IR band is observed in the range of 1400–1600 cm^−1^ owing to aromatic C=C stretching vibrations. The corresponding spectrum of Fe_3_O_4_@*Fritillaria* NPs is depicted in Fig. 2c. It is literally a combination of Fig. 2a,b indicating the successful functionalization of *Fritillaria* molecules over the ferrite NPs. These biomolecules actually perform as excellent capping agent, preventing the NPs from agglomeration and oxidation^52^. It also acts as reducing and stabilizing agent for immobilizing Pd NPs on the ferrite surface. The FT-IR spectrum of Fe_3_O_4_@*Fritillaria*/Pd NPs (Fig. 2d) is almost alike Fig. 2c except a small shift in C‒O, C=C and O‒H stretching frequencies. These shifts account for the attachment of Pd NPs on the surface modified MNPs.

**Figure 3:**
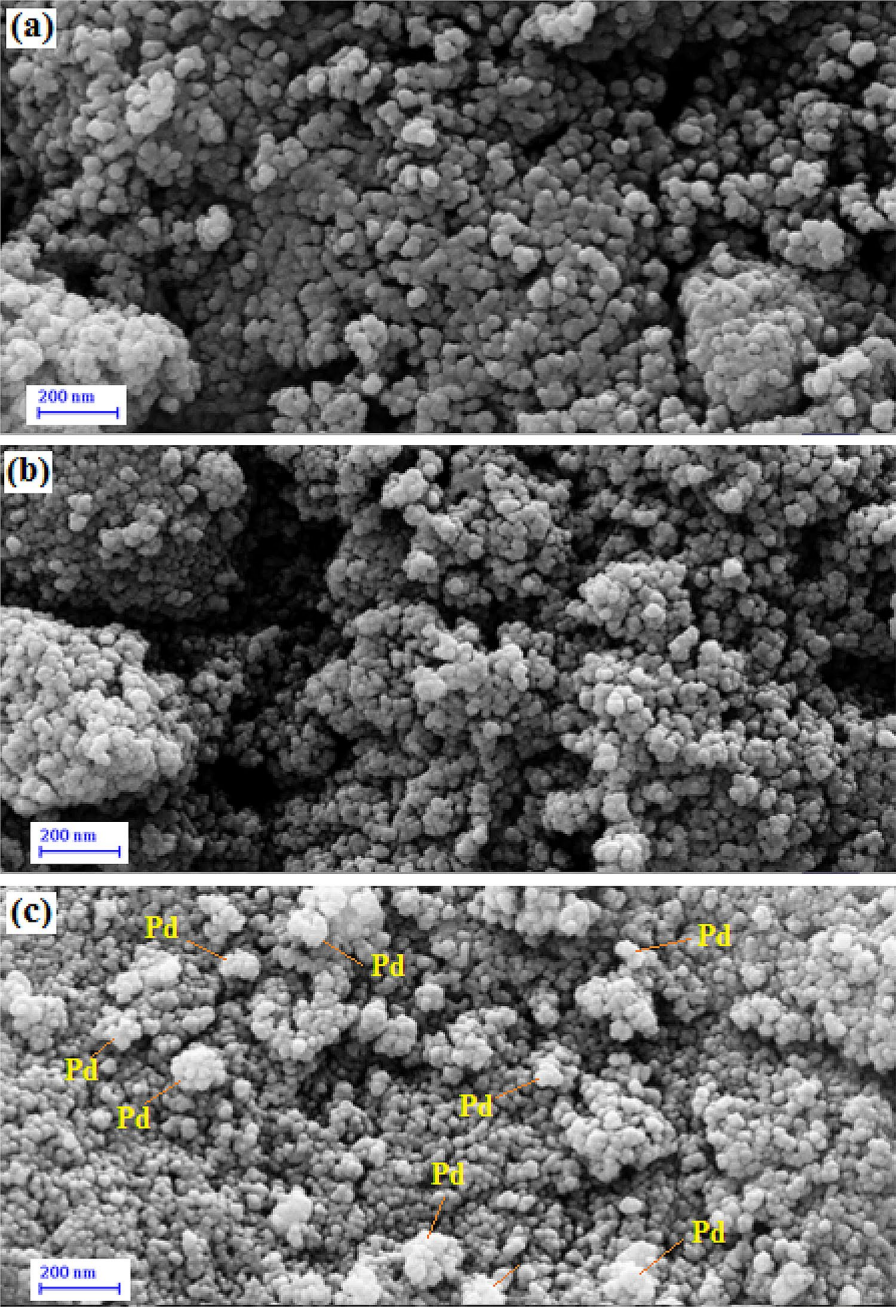
FE-SEM images of the (**a**) Fe_3_O_4_ NPs, (**b**) Fe_3_O_4_@*Fritillaria* NPs, (**c**) Fe_3_O_4_@*Fritillaria*/PdNPs.

The structural morphology, size and shape of the Fe_3_O_4_, Fe_3_O_4_@*Fritillaria*, and Fe_3_O_4_@*Fritillaria*/Pd nanocomposite were investigated with the FE-SEM analysis as shown in Fig. 3. The materials are of nanometric size and of quasi-spherical shape (Fig. 3a). In addition, a continuous biopolymer layer is seen on the nanocomposite surface indicating the surface modification (Fig. 3b,c). The bright spots in Fig. 3c signifies the in situ synthesized Pd NPs being spread over the Fe_3_O_4_@*Fritillaria* composite.

**Figure 4:**
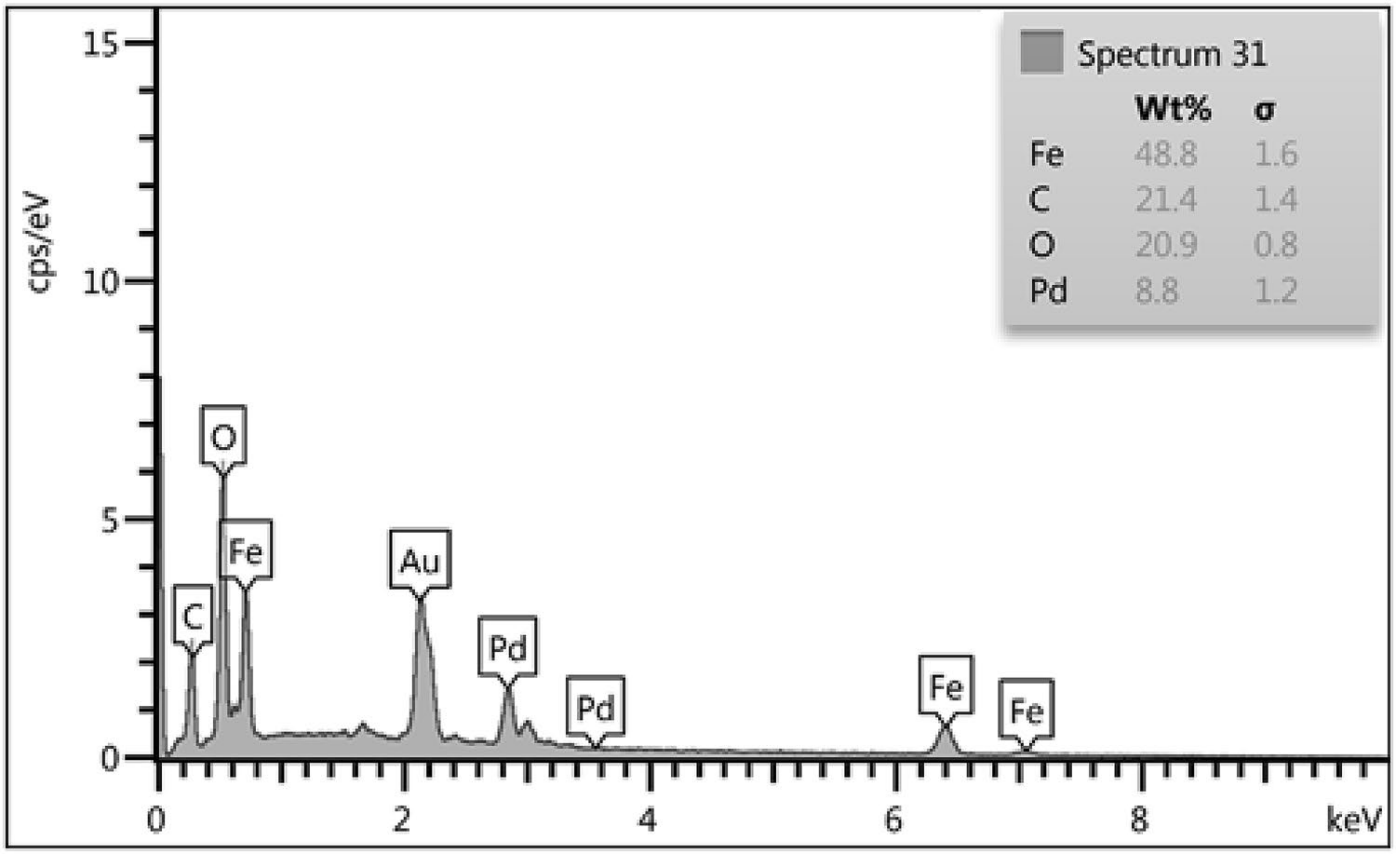
EDX of the Fe_3_O_4_@*Fritillaria*/Pd NPs.

EDX analysis of the material was conducted in order to know the chemical composition. The spectrum obtained on recording of signals at random points of the catalyst surface showed the presence of Fe, Pd as metallic and C, O as non-metallic components. The non metals justify the attachment of phyto-compounds in the composite (Fig. 4).

**Figure 5:**
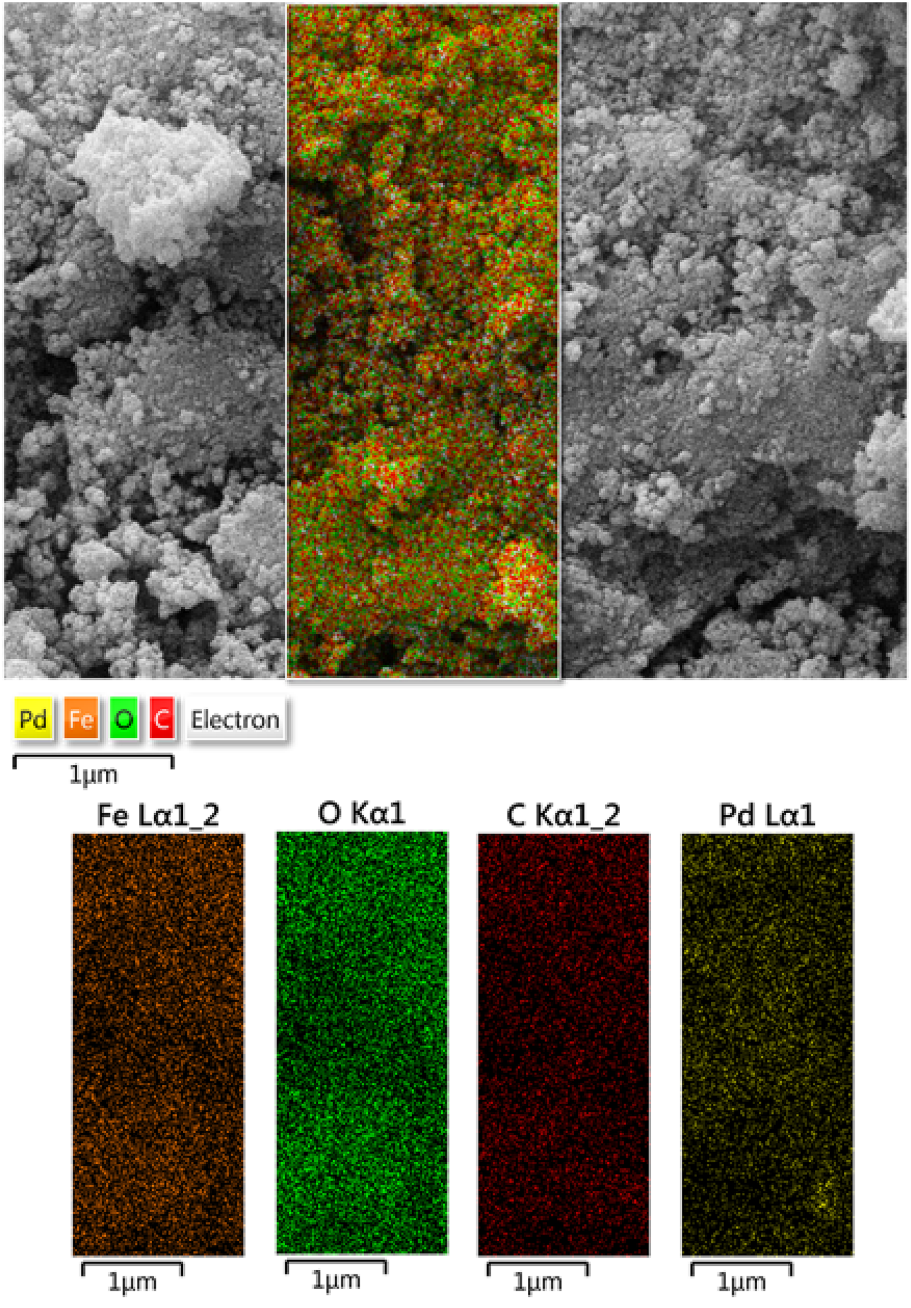
Elemental mapping of Fe_3_O_4_@*Fritillaria*/Pd nanocomposite.

In addition to the EDX analysis, elemental mapping of Fe_3_O_4_@*Fritillaria*/Pd nanocomposite also carried out to have the knowledge of component distributions over the catalyst surface. X-ray scanning of a segment of the FE-SEM image reveals the homomorphic dispersion of all the components on the nanocomposite (Fig. 5). The uniform distribution of the active site definitely has a significant role behind its catalytic superiority.

**Figure 6:**
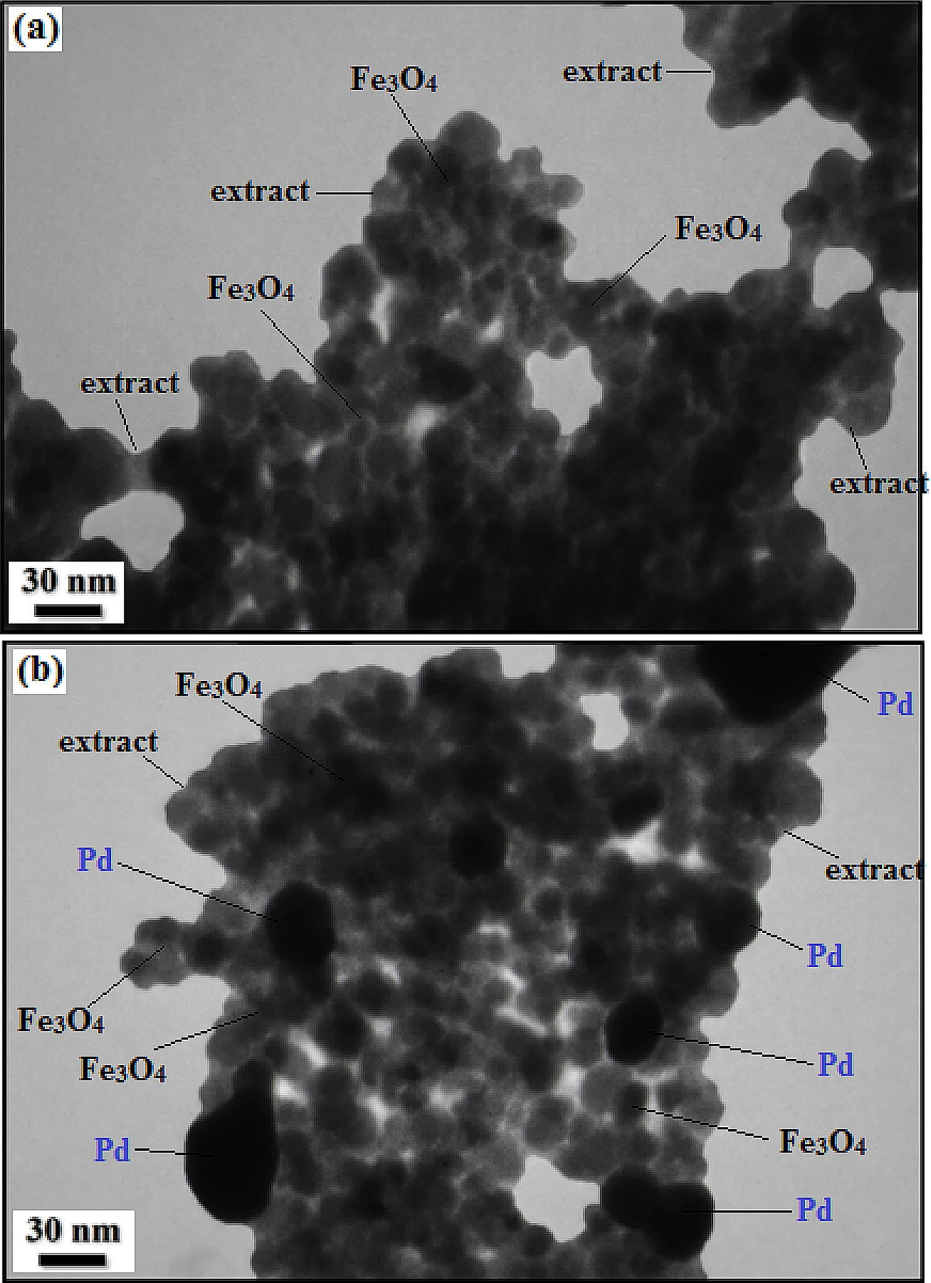
TEM images of (**a**) Fe_3_O_4_@*Fritillaria* NPs and (**b**) Fe_3_O_4_@*Fritillaria*/Pd NPs.

The TEM image of the Fe_3_O_4_@*Fritillaria*/Pd NPs exhibits that the Pd NPs are formed with almost globular morphology (Fig. 6). As can be seen in the image (Fig. 6a), the ferrite NPs are of 10–20 nm in dimension that are coated by thin layers of *Fritillaria* extract. The biomolecular layers from the extract acts as the green reducing agent of the Pd ions as well as the stabilizing agent of Pd NPs. It easily detectable that the dark Pd NPs are of ~ 20–30 nm being entrapped in the modified iron oxide surface (Fig. 6b).

**Figure 7:**
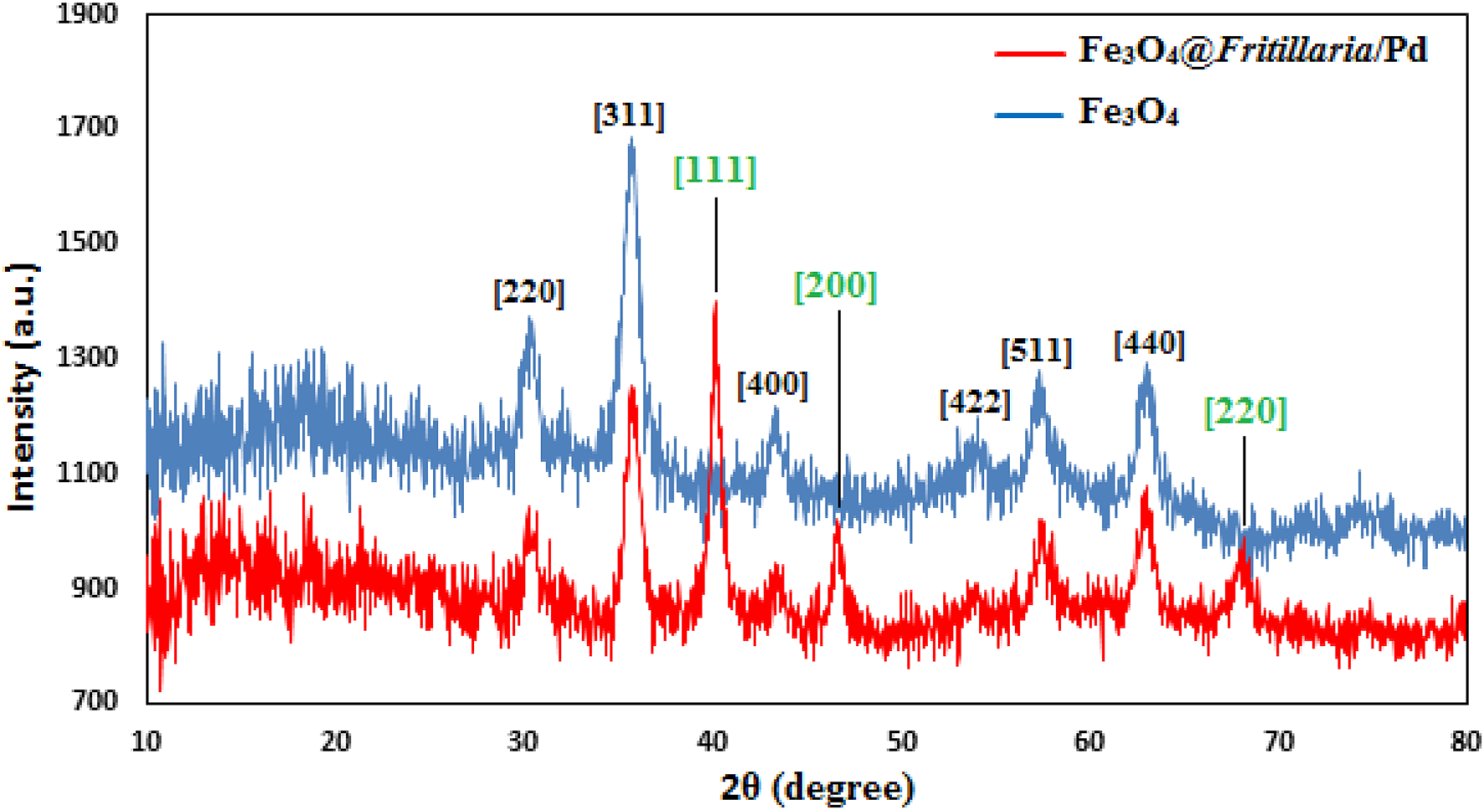
XRD profile of Fe_3_O_4_ NPs and the Fe_3_O_4_@*Fritillaria*/Pd nanocomposite.

Figure 7 illustrates XRD patterns of Fe_3_O_4_ and Fe_3_O_4_@*Fritillaria*/Pd nanocomposite. Evidently, XRD profile of the latter carries all the significant peaks that of cubic spinel Fe_3_O_4_ NPs. The XRD peaks found at 2θ = 30.3°, 35.7°, 43.4°, 53.9°, 57.4° and 62.9° can be attributed to diffraction on (220), (311), (400), (422), (511) and (440) planes respectively (JCPDS No. 19-0629). It also implies that the interior structure remains undisturbed even after bio-functionalizations and Pd anchoring. The Pd attachment can also be demonstrated by the distinctive peaks observed at 2θ = 40.1°, 46.6° and 67.9°, being ascribed to the (111), (200) and (220) crystalline planes of Pd *fcc* structure.

**Figure 8:**
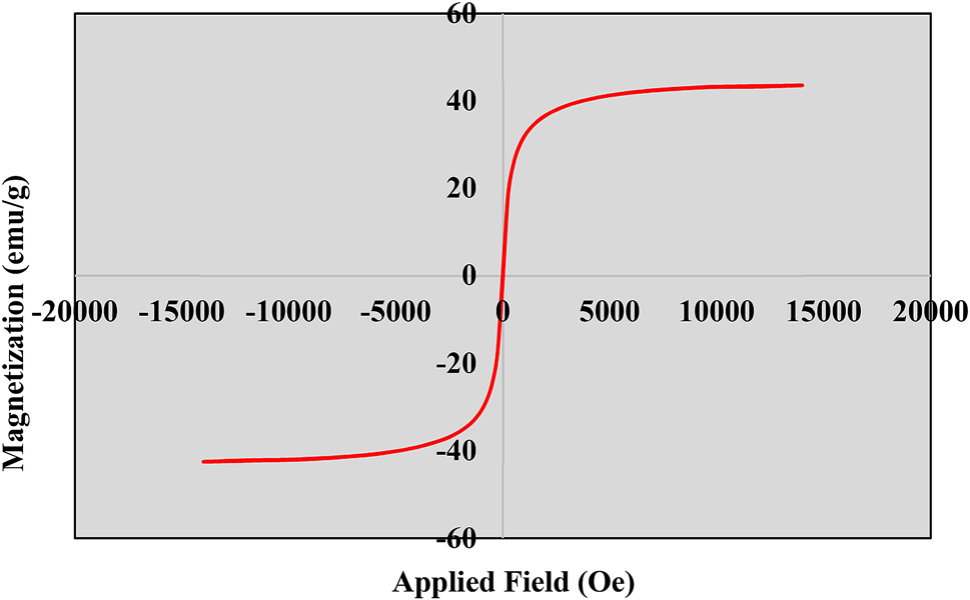
Magnetism study of Fe_3_O_4_@*Fritillaria*/Pd NPs.

Magnetic characteristics of the Fe_3_O_4_@*Fritillaria*/Pd NPs was assessed through VSM analysis and the magnetization curve has been shown in Fig. 8. From the corresponding hysteris curve, the maximal saturation magnetization of Fe_3_O_4_@*Fritillaria*/Pd NPs was found to appear at 42.5 emu g^−1^. However, the value is much lower than bare ferrite NP (64.2 emu g^−1^) due to surface operations. Still, in the modified material the magnetization goes down from plateau state to zero on removal of the magnetic field which justifies it to be superparamagnetic^53^.

### Catalytic applications of Fe_3_O_4_@***Fritillaria***/Pd nanocomposite

So as to explore the catalytic application of Fe_3_O_4_@*Fritillaria*/Pd nanocomposite and finding optimum reaction conditions, we selected the reduction of nitrobenzene as a model reaction. The effect of various conditions including temperature, solvents, catalyst load, amount of reductant and time reaction were studied over the reaction. The outcomes were documented in Table 1. Primarily, the model reaction was examined in various solvents like dimethyl formamide (DMF), EtOH, MeOH, CH_3_CN, H_2_O/EtOH and H_2_O. Among them, H_2_O/EtOH afforded the best yield and thereby selected as the optimum solvent. The amount ofFe_3_O_4_@*Fritillaria*/Pd nanocomposite was also explored for the model reaction. Based on the study, 0.1 mol% catalyst was the most convenient for 1 mmol of nitrobenzene. Finally, the best result for the reduction of nitrobenzene was obtained using 3.0 mmol NH_2_NH_2_·H_2_O as reductant and 0.013 g Fe_3_O_4_@*Fritillaria*/Pd (0.1 mol% Pd) catalyst respectively inH_2_O/EtOH (2:1) solvent at 80 °C. We also performed the reduction of nitrobenzene using the bare Fe_3_O_4_@*Fritillaria* NPs but only a trace of aniline was obtained. This indicates that the interaction between the Pd NPs and Fe_3_O_4_@Fritillaria is very important for catalytic success.

**Table 1:** Standardization of reaction conditions in the reduction of nitrobenzene over Fe_3_O_4_@*Fritillaria*/Pd nanocomposite^a^.


Entry	Catalyst (mol%)	Solvent	N_2_H_4_·H_2_O (mmol)	Condition	Time (h)	Yield (%)^b^
1	–	EtOH	3	Reflux	24	0
2	0.1	EtOH	3	Reflux	2	75
3	0.1	MeOH	3	Reflux	2	70
4	0.1	H_2_O	3	Reflux	6	60
5	0.1	DMF	3	Reflux	2	55
6	0.1	CH_3_CN	3	Reflux	2	50
7	0.1	H_2_O/EtOH (1:1)	3	Reflux	1	90
8	0.1	H_2_O/EtOH (2:1)	3	Reflux	0.5	98
9	0.05	H_2_O/EtOH (2:1)	3	Reflux	1	85
10	0.1	H_2_O/EtOH (2:1)	2.5	Reflux	1	90
11	0.1	H_2_O-EtOH (2:1)	3.5	Reflux	0.5	98
12	0.1	H_2_O/EtOH (2:1)	3	r.t	2	45

^a^Reaction conditions: nitrobenzene (1.0 mmol), solvent (3.0 mL), open air; '^b^Isolated yield.

After resolving the required optimizations, the next endeavor was to generalize them over a range of differently functionalized (electron-donating and electron-withdrawing groups) nitroarenes. The results in terms of reaction yield and TOF are shown in Table 2. All the reactions were executed superbly with all kind of substrates without noticeable influence of functional groups on the reaction. All the reactions were completed within 0.5–2 h.

**Table 2:** Reduction of aromatic nitroarenes catalyzed by Fe_3_O_4_@*Fritillaria*/Pd NPs^a^.

Entry	RC_6_H_4_NO_2_	Time (h)	Yield (%)^b^	TOF (10^−3^) (s^−1^)^c^
1	H	0.5	98	544
2	4-OH	0.5	98	544
3	2-OH	1	92	256
4	4-NH_2_	1	95	264
5	4-CH_3_	0.5	96	533
6	4-OCH_3_	0.5	95	528
7	4-CN	1	92	256
8	2-NH_2_	2	88	122
9	4-CHO	1.5	85	157
10	4-Cl	1.5	80	148

^a^Reaction conditions: Nitroarene (1.0 mmol), NH_2_NH_2_. H_2_O (3.0 mmol), catalyst (0.1 mol%), EtOH:H_2_O (1:2, 3.0 mL), 80 °C; ^b^Isolated yields; ^c^turnover frequencies (TOF = (Yield/Time)/Amount of catalyst (mol).

### Recyclability of Fe_3_O_4_@***Fritillaria***/Pd catalyst

**Figure 9:**
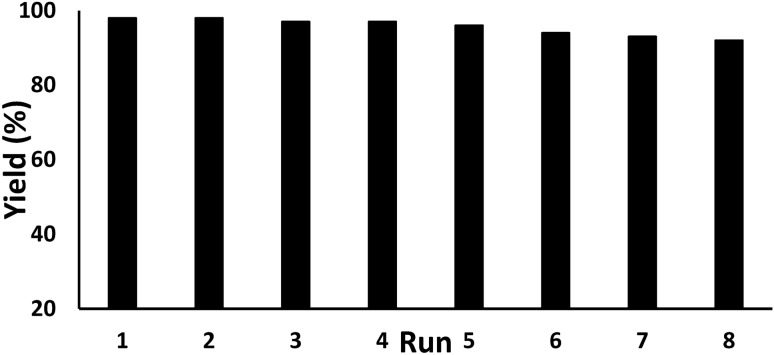
Reusability of Fe_3_O_4_@*F*ritillaria/Pd catalyst for reduction of nitrobenzene.

**Figure 10:**
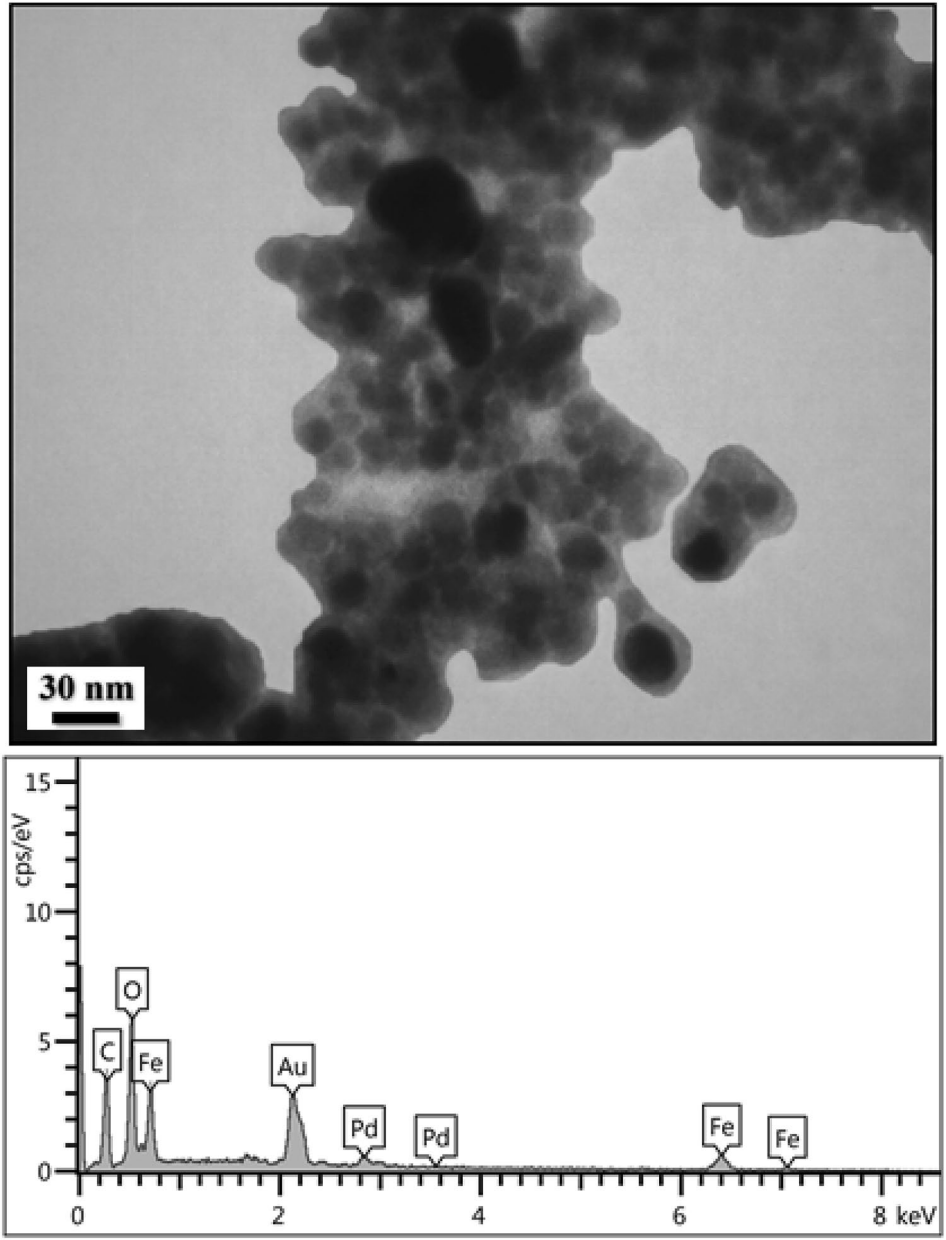
TEM and EDX data for reused Fe_3_O_4_@*Fritillaria*/Pd catalyst after 7 runs.

For every heterogeneous catalytic system, the isolation and recycling of catalyst is a crucial feature in view of sustainable and industrial concern. The reusability of Fe_3_O_4_@*Fritillaria*/Pd was examined over the reduction of nitrobenzene under optimized conditions. After finishing a fresh batch of reaction the catalyst was recovered using a bar magnet and washed several times with ethanol and water. It was regenerated after drying at moderate temperature. To our delight, we could have reused it for eight consecutive cycles of reaction without noticeable loss in its activity (Fig. 9). We further analyzed the structural morphology of Fe_3_O_4_@*Fritillaria*/Pd nanocomposite after recycling 7 times by using TEM and EDX. As clearly can be seen from the TEM image (Fig. 10), the catalyst retains its initial morphology and particles size without any sign of agglomeration. Alongside, there occurs no change in elemental composition as evident from EDX data (Fig. 10), which in turn validates the robustness of our material.

### Heterogeneity test for Fe_3_O_4_@***Fritillaria***/Pd catalyst

**Figure 11:**
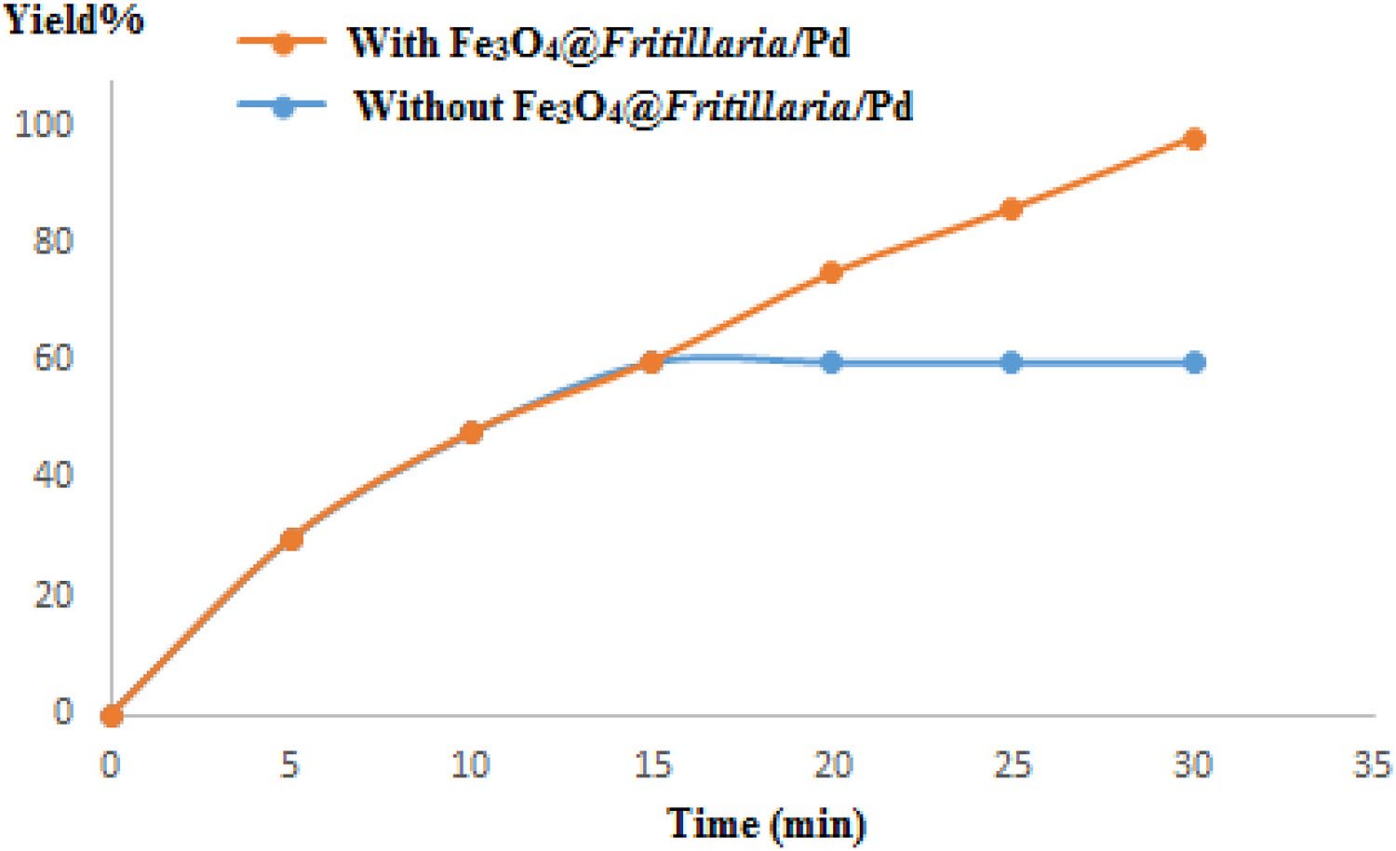
Hot filtration and leaching test of Fe_3_O_4_@*Fritillaria*/Pd in the reduction of nitrobenzene.

The Sheldon's test was carried out to assure heterogeneous nature of the synthesized material, whether any Pd species leached out in the filtrate solution. The reduction of nitrobenzene was continued over the catalyst under optimized state for 15 min and then the reaction mixture was divided into two-halves. From one portion of the reaction mixture the catalyst was removed by a magnetic bar and both the part reactions were further continued for another 15 min. On gas chromatographic analysis, it was revealed that no significant progress in reaction was achieved under non-catalytic conditions (60% conversion) while the other portion leaded to completion. The result is shown in Fig. 11. This further suggests that there was hardly any leaching of Pd NPs took place in the reaction mixture justifying its true heterogeneity.

### Study of reaction mechanism

**Scheme 2:**
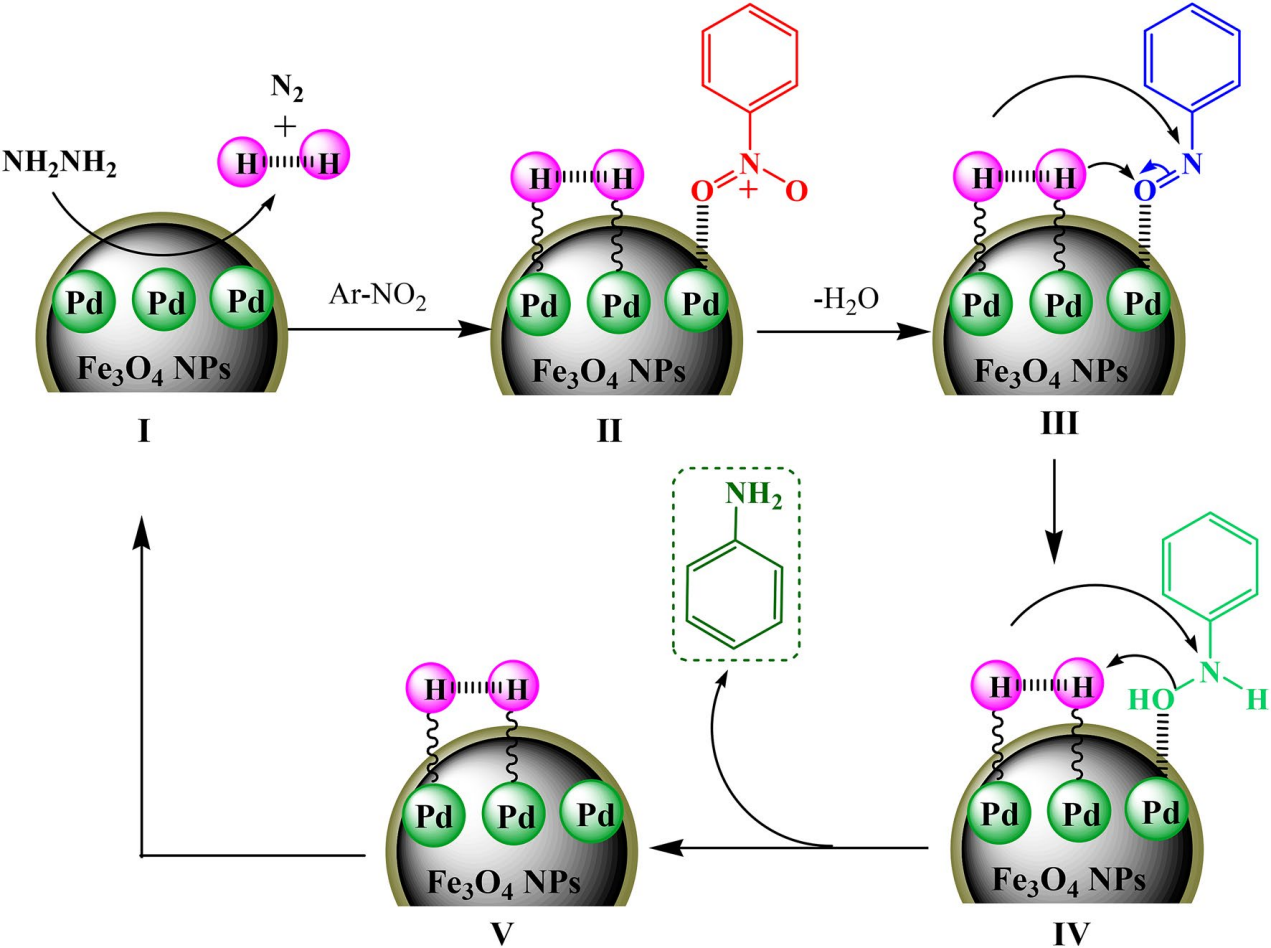
Reaction mechanism for the reduction of nitrobenzene over Fe_3_O_4_@*Fritillaria*/Pd catalyst.

Based on earlier published works, a probable reaction pathway has been documented in Scheme 2^54–57^. The reaction goes through several intermediates. At the outset, hydrazine gets adsorbed on the surface of Pd NPs (I) which subsequently generates N_2_ and nascent hydrogen by bond cleavage. This hydrogen is captured by the active Pd NPs to form metal hydride (II). In the meantime the substrate nitroarenes also get adsorbed over the catalyst surface and gets reduced by hydride transfer from II to form active nitroso derivative (III). This moiety is then further reduced to amine via hydroxylamine (IV) intermediate through hydrogen transfer. The hydrogenation of hydroxylamine is considered to be slow and rate determining step. Finally, the desired product leaves behind the catalyst surface to be used for the next cycle.

### Uniqueness of our result

The individuality of our protocol was affirmed by comparing the catalytic performance between our methodology and the reported procedures in the reduction of 4‐nitrophenol. The results are shown in Table 3 which evidently displays that the Fe_3_O_4_@*Fritillaria*/Pd nanocomposite is much superior to others in terms of reaction time and yield.

**Table 3:** Catalytic comparison in the reduction of 4-nitrophenol.

Entry	Catalyst (mol%)	Conditions	Time (h)	Yield (%)	References
1	Au/MTA	NaBH_4_, EtOH, RT	3	90	^58^
2	Fe_3_O_4_ Ni MNPs	Glycerol, KOH, 80 °C	3.5	88	^59^
3	Pd NPs/RGO	NaBH_4_, EtOH:H_2_O, 50 °C	1.5	97	^60^
4	Fe–phenanthroline/C	N_2_H_4_. H_2_O, THF, 100 °C	10	97	^61^
5	Nickel–iron mixed oxide	N_2_H_4_. H_2_O, propan-2-ol, Reflux	1.75	93	^62^
6	[Pt]@SiC_6_	AcOEt, H_2_, RT	3	99	^63^
7	Rh	N_2_H_4_, EtOH, 80 °C	2.5	94	^64^
8	Rh–Fe_3_O_4_ nanocrystals	N_2_H_4_, EtOH, 80 °C	1	99	^65^
9	PdCu/graphene	NaBH_4_, EtOH:H_2_O (1:2), 50 °C	1.5	98	^66^
10	Fe_3_O_4_@Fritillaria/Pd	N_2_H_4_. H_2_O, EtOH:H_2_O (1:2), 80 °C	0.5	98	This work

## Conclusion

We introduce a facile procedure for the synthesis of a heterogeneous and reusable Pd NPs decorated on *Fritillaria imperialis* flower extract modified magnetic ferrite nanoparticles by post functionalization approach. Catalytic performance of the Fe_3_O_4_@*Fritillaria*/Pd nanocomposite material was studied in the competent reduction of nitroarenes without the use of any added base. The protocol worked proficiently using hydrazine hydrate as the reducing agent under eco-friendly conditions affording various aromatic amines with excellent yields. In addition, due to strong magnetic nature, the Fe_3_O_4_@*Fritillaria*/Pd nanocatalyst could be reused as much as eight cycles in the reduction process emphasizing its true heterogeneity. In view of the outstanding catalytic behavior, the engineered material is anticipated to be a versatile support to feed many other noble metals like Ag, Au, Cu etc. towards many catalytic transformations and might find an excellent exposure in chemical industry.

## References

[1] Xu C, Nasrollahzadeh M, Sajjadi M, Maham M, Luque R, Puente-Santiago AR (2019). Benign-by-design nature-inspired nanosystems in biofuels production and catalytic applications. Renew. Sust. Energy Rev..

[2] Baig RBN, Varma RS (2013). Copper on chitosan: a recyclable heterogeneous catalyst for azide–alkyne cycloaddition reactions in water. Green Chem..

[3] Kainz QM, Reiser O (2014). Polymer-and dendrimer-coated magnetic nanoparticles as versatile supports for catalysts, scavengers, and reagents. Acc. Chem. Res..

[4] Liu J, Chen L, Cui H, Zhang J, Zhang L, Su CY (2014). Applications of metal–organic frameworks in heterogeneous supramolecular catalysis. Chem. Soc. Rev..

[5] Guo W, Jiao J, Tian K, Tang Y, Jia Y, Li R, Xu Z, Wang H (2015). Controllable synthesis of core–satellite Fe_3_O_4_@polypyrrole/Pd nanoarchitectures with aggregation-free Pd nanocrystals confined into polypyrrole satellites as magnetically recoverable and highly efficient heterogeneous catalysts. RSC Adv..

[6] Chen F, Chen A (2014). A facile one-pot route to one-dimensional Fe_3_O_4_–polypyrrolenanocomposites. Chem. Lett..

[7] Veisi H, Sarachegol P, Hemmati S (2018). Palladium(II) anchored on polydopamine coated-magnetic nanoparticles (Fe_3_O_4_@PDA@Pd(II)): a heterogeneous and core–shell nanocatalyst in Buchwald–Hartwig C–N cross coupling reactions. Polyhedron.

[8] Baig RBN, Varma RS (2013). Magnetically retrievable catalysts for organic synthesis. Chem. Commun..

[9] Dalpozzo R (2015). Magnetic nanoparticle supports for asymmetric catalysts. Green Chem..

[10] Nemati M, Tamoradi T, Veisi H (2019). Mobilization of Gd (III) complex on Fe_3_O_4_: a novel and recyclable catalyst for synthesis of tetrazole and S–S coupling. Polyhedron.

[11] Nasrollahzadeh M, Issaabadi Z, Sajadi SM (2018). Fe3O4@SiO2 nanoparticle supported ionic liquid for green synthesis of antibacterially active 1-carbamoyl-1-phenylureas in water. RSC Adv..

[12] Gawande MB, Branco PS, Varma RS (2013). Nano-magnetite (Fe_3_O_4_) as a support for recyclable catalysts in the development of sustainable methodologies. Chem. Soc. Rev..

[13] Pagoti S, Ghosh T, Dash J (2016). Synthesis of magnetic nanoparticles and polymer supported imidazolidinone catalysts for enantioselective friedel-crafts alkylation of indoles. Chemistry Select.

[14] Narollahzadeh M, Issaabadi Z, Varma RS (2019). Magnetic lignosulfonate-supported Pd complex: renewable resource-derived catalyst for aqueous Suzuki-Miyaura reaction. ACS Omega.

[15] Kadam RG, Rathi AK, Zboril R, Varma RS, Gawande MB, Jayaram RV (2017). Hexagonal mesoporous silica-supported copper oxide (CuO/HMS) catalyst: synthesis of primary amides from aldehydes in aqueous medium. ChemPlusChem.

[16] Astruc D, Lu F, Aranzaes JR (2005). Nanoparticles as recyclable catalysts: the frontier between homogeneous and heterogeneous catalysis. Angew. Chem. Int. Ed..

[17] Murray CB, Kagan CR, Bawendi MG (2000). Synthesis and characterization of monodisperse nanocrystals and close-packed nanocrystal assemblies. Annu. Rev. Mater. Sci..

[18] Zhang K, Suh JM, Choi J-W, Jang HW, Shokouhimehr M, Varma RS (2019). Recent advances in the nanocatalyst-assisted NaBH_4_ reduction of nitroaromatics in water. ACS Omega.

[19] Kharissova OV, Rasika Dias HV, Kharisov BI, Olvera Perez B, Jimenez Perez VM (2013). The greener synthesis of nanoparticles. Trends Biotechnol..

[20] Hoag GE, Collins JB, Holcomb JL, Hoag JR, Nadagouda MN, Varma RS (2009). Degradation of bromothymol blue by 'Greener' nano-scale zero-valent iron synthesized using tea polyphenols. J. Mater. Chem..

[21] Plachtová P, Medříková Z, Zbořil R, Tuček J, Varma RS, Maršálek B (2018). Iron and iron oxide nanoparticles synthesized using green tea extract: improved ecotoxicological profile and ability to degrade malachite green. ACS Sustain. Chem. Eng..

[22] Nadagouda MN, Castle AB, Murdock RC, Hussain SM, Varma RS (2010). In vitro biocompatibility of nanoscale zerovalent iron particles (NZVI) synthesized using tea polyphenols. Green Chem..

[23] Markova Z, Novak P, Kaslik J, Plachtova P, Brazdova M, Jancula D, Siskova KM, Machala L, Marsalek B, Zbořil R, Varma RS (2014). Iron(II, III)-polyphenol complex nanoparticles derived from green tea with remarkable ecotoxicological impact. ACS Sustain. Chem. Eng..

[24] Smuleac V, Varma R, Sikdar S, Bhattacharyya D (2011). Green synthesis of Fe and Fe/Pd bimetallic nanoparticles in membranes for reductive degradation of chlorinated organics. J. Membrane Sci..

[25] Kumar KM, Mandal BK, Kumar KS, Reddy PS, Sreedhar B (2013). Biobased green method to synthesise palladium and iron nanoparticles using *Terminalia chebula* aqueous extract. Spectrochim. Acta A.

[26] Veisi H, Safarimehr P, Hemmati S (2019). Buchwald–Hartwig C–N cross coupling reactions catalyzed by palladium nanoparticles immobilized on thio modified-multi walled carbon nanotubes as heterogeneous and recyclable nanocatalyst. Appl. Organomet. Chem..

[27] Veisi H, Tamoradi T, Rashtiani A, Hemmati S, Karmakar B (2020). Palladium nanoparticles anchored polydopamine-coated graphene oxide/Fe_3_O_4_ nanoparticles (GO/Fe_3_O_4_@PDA/Pd) as a novel recyclable heterogeneous catalyst in the facile cyanation of haloarenes using K_4_[Fe(CN)_6_] as cyanide source. J. Ind. Eng. Chem..

[28] Dewan A, Sarmah M, Thakur AJ, Bharali P, Bora U (2018). Greener biogenic approach for the synthesis of palladium nanoparticles using papaya peel: an eco-friendly catalyst for C–C coupling reaction. ACS Omega.

[29] Ko YL, Krishnamurthy S, Yun YS (2015). Facile synthesis of monodisperse Pt and Pd nanoparticles using antioxidants. J. Nanosci. Nanotechnol..

[30] Sarmah M, Neog AB, Boruah PK, Das MR, Bharali P, Bora U (2019). Effect of substrates on catalytic activity of biogenic palladium nanoparticles in C–C cross-coupling reactions. ACS Omega.

[31] Vishnukumar P, Vivekanandhan S, Muthuramkumar S (2017). Plant-mediated biogenic synthesis of palladium nanoparticles: recent trends and emerging opportunities. ChemBioEng Rev..

[32] Kharissova OV, Dias HVR, Kharisov BI, Pérez BO, Pérez VM (2013). The greener synthesis of nanoparticles. J. Biotechnol..

[33] Abboud Y, Saffaj T, Chagraoui A, El Bouari A, Brouzi K, Tanane O, Ihssane B (2014). Biosynthesis, characterization and antimicrobial activity of copper oxide nanoparticles (CONPs) produced using brown alga extract (*Bifurcaria bifurcata*). Appl. Nanosci..

[34] Veisi H, Ghorbani M, Hemmati S (2019). Sonochemical in situ immobilization of Pd nanoparticles on green tea extract coated Fe3O4 nanoparticles: an efficient and magnetically recyclable nanocatalyst for synthesis of biphenyl compounds under ultrasound irradiations. Mater. Sci. Eng. C.

[35] Veisi H, Mohammadi L, Hemmati S, Tamoradi T, Mohammadi P (2019). In Situ immobilized silver nanoparticles on *Rubia tinctorum* extract-coated ultrasmall iron oxide nanoparticles: an efficient nanocatalyst with magnetic recyclability for synthesis of propargylamines by A3 coupling reaction. ACS Omega.

[36] Shahriary M, Veisi H, Hekmati M, Hemmati S (2018). In situ green synthesis of Ag nanoparticles on herbal tea extract (*Stachys lavandulifolia*)-modified magnetic iron oxide nanoparticles as antibacterial agent and their 4-nitrophenol catalytic reduction activity. Mater. Sci. Eng. C.

[37] Veisi H, Moradi SB, Saljooqi A, Safarimehr P (2019). Silver nanoparticle-decorated on tannic acid-modified magnetite nanoparticles (Fe3O4@TA/Ag) for highly active catalytic reduction of 4-nitrophenol, Rhodamine B and Methylene blue. Mater. Sci. Eng. C.

[38] Veisi H, Ghorbani F (2017). Iron oxide nanoparticles coated with green tea extract as a novel magnetite reductant and stabilizer sorbent for silver ions: synthetic application of Fe_3_O_4_@ green tea/Ag nanoparticles as magnetically separable and reusable nanocatalyst for reduction of 4-nitrophenol. Appl. Organomet. Chem..

[39] Hemmati S, Heravi MM, Karmakar B, Veisi H (2020). Green fabrication of reduced graphene oxide decorated with Ag nanoparticles (rGO/Ag NPs) nanocomposite: a reusable catalyst for the degradation of environmental pollutants in aqueous medium. J. Mol. Liquid..

[40] Veisi H, Hemmati S, Shirvani H, Veisi H (2016). Green synthesis and characterization of monodispersed silver nanoparticles obtained using oak fruit bark extract and their antibacterial activity. Appl. Orgmetal. Chem..

[41] Datta KJ, Rathi AJ, Gawande MB, Ranc V, Zoppellaro G, Varma RS, Zboril R (2016). Base-free transfer hydrogenation of nitroarenes catalyzed by micro-mesoporous iron oxide. ChemCatChem.

[42] Datta KJ, Rathi AK, Kumar P, Kaslik J, Medrik I, Ranc V, Varma RS, Zboril R, Gawande MB (2017). Synthesis of flower-like magnetite nanoassembly: application in the efficient reduction of nitroarenes. Sci. Rep..

[43] Hira SA, Nallal M, Park KH (2019). Fabrication of PdAg nanoparticle infused metal-organic framework for electrochemical and solution-chemical reduction and detection of toxic 4-nitrophenol. Sens. Actuators: B Chem..

[44] Hira SA, Hui HS, Yousuf M, Park KH (2020). Silver nanoparticles deposited on metal tungsten bronze as a reusable catalyst for the highly efficient catalytic hydrogenation/reduction of 4-nitrophenol. Catal. Commun..

[45] Ayad MM, Amer WA, Kotp MG (2017). Magnetic polyaniline-chitosan nanocomposite decorated with palladium nanoparticles for enhanced catalytic reduction of 4-nitrophenol. Mol. Catal..

[46] Eichenbaum G, Johnson M, Kirkland D, O’Neill P, Stellar S, Bielawne J, DeWire R, Areia D, Bryant S, Weiner S, Desai-Krieger D (2009). Assessment of the genotoxic and carcinogenic risks of p-nitrophenol when it is present as an impurity in a drug product. Regul. Toxicol. Pharmacol..

[47] Ju KS, Parales RE (2010). Nitroaromatic compounds, from synthesis to biodegradation. Microbiol. Mol. Biol. Rev..

[48] Shah MT, Balouch A, Pathan AA, Mahar AM, Sabir S, Khattak R, Umar AA (2017). SiO_2_ caped Fe_3_O_4_ nanostructures as an active heterogeneous catalyst for 4-nitrophenol reduction. Microsyst. Technol..

[49] Rolim WR, Pelegrino MT, de Araújo Lima B, Ferraz LS, Costa FN, Bernardes JS, Rodigues T, Brocchi M, Seabra AB (2019). Green tea extract mediated biogenic synthesis of silver nanoparticles: characterization, cytotoxicity evaluation and antibacterial activity. Appl. Surf. Sci..

[50] Ahluwalia V, Elumalai S, Kumar V, Kumar S, Sangwan RS (2018). Nano silver particle synthesis using *Swertia paniculata* herbal extract and its antimicrobial activity. Microb. Pathog..

[51] Banumathi B, Vaseeharan B, Suganya P, Citarasu T, Govindarajan M, Alharbi NS, Kadaikunnan S, Khaled JM, Benelli G (2017). Toxicity of Camellia sinensis-fabricated silver nanoparticles on invertebrate and vertebrate organisms: morphological abnormalities and DNA damages. J. Clust. Sci..

[52] Kim DK, Mikhaylova M, Zhang Y, Muhammed M (2003). Protective coating of superparamagnetic iron oxide nanoparticles. Chem. Mater..

[53] Karimzadeh I, Aghazadeh M, Doroudi T, Ganjali MR, Kolivand PH (2017). Superparamagnetic iron oxide (Fe_3_O_4_) nanoparticles coated with PEG/PEI for biomedical applications: a facile and scalable preparation route based on the cathodic electrochemical deposition method. Adv. Phys. Chem..

[54] Petkar DR, Kadu BS, Chikate RC (2014). Highly efficient and chemoselective transfer hydrogenation of nitroarenes at room temperature over magnetically separable Fe–Ni bimetallic nanoparticles. RSC Adv..

[55] El-Hout SI, El-Sheikh SM, Hassan HMA, Harraz FA, Ibrahim IA, El-Sharkawy EA (2015). A green chemical route for synthesis of graphene supported palladium nanoparticles: a highly active and recyclable catalyst for reduction of nitrobenzene. Appl. Catal. A: Gen..

[56] Zuo Y, Song J-M, Niu H-L, Mao C-J, Zhang S-Y, Shen Y-H (2016). Synthesis of TiO_2_-loaded Co0.85Se thin films with heterostructure and their enhanced catalytic activity for p-nitrophenol reduction and hydrazine hydrate decomposition. Nanotechnology.

[57] Li M, Chen G (2013). Revisiting catalytic model reaction p-nitrophenol/NaBH_4_ using metallic nanoparticles coated on polymeric spheres. Nanoscale.

[58] Fountoulaki S, Daikopoulou V, Gkizis PL, Tamiolakis I, Armatas GS, Lykakis IN (2014). Mechanistic studies of the reduction of nitroarenes by NaBH_4_ or hydrosilanescatalyzed by supported gold nanoparticles. ACS Catal..

[59] Gawande MB, Rathi AK, Branco PS, Nogueira ID, Velhinho A, Shrikhande JJ, Indulkar UU, Jayaram RV, Ghumman CAA, Bundaleski N, Teodoro OMND (2012). Regio and chemoselective reduction of nitroarenes and carbonyl compounds over recyclable magnetic ferrite–nickel nanoparticles (Fe_3_O_4_–Ni) by using glycerol as a hydrogen source. Chem. Eur. J..

[60] Nasrollahzadeh M, Sajadi SM, Rostami-Vartooni A, Alizadeh M, Bagherzadeh M (2016). Green synthesis of the Pd nanoparticles supported on reduced graphene oxide using barberry fruit extract and its application as a recyclable and heterogeneous catalyst for the reduction of nitroarenes. J. Colloid Interface Sci..

[61] Jagadeesh RV, Wienhofer G, Westerhaus FA, Surkus A-E, Pohl M-M, Junge H, Junge K, Beller M (2011). Efficient and highly selective iron-catalyzed reduction of nitroarenes. Chem. Commun..

[62] Shi Q, Lu R, Lu L, Fu X, Zhao D (2007). Efficient reduction of nitroarenes over nickel–iron mixed oxide catalyst prepared from a nickel–iron hydrotalciteprecursor. Adv. Synth. Catal..

[63] Motoyama Y, Kamo K, Nagash H (2009). Catalysis in polysiloxane gels: platinum-catalyzed hydrosilylation of polymethylhydrosiloxane leading to reusable catalysts for reduction of nitroarenes. Org. Lett..

[64] Shokouhimehr M, Lee JE, Han SI, Hyeon T (2013). Magnetically recyclable hollow nanocomposite catalysts for heterogeneous reduction of nitroarenes and Suzuki reactions. Chem. Commun..

[65] Jang Y, Kim S, Jun SW, Kim BH, Hwang S, Song IK, Kim BM, Hyeon T (2011). Simple one-pot synthesis of Rh-Fe_3_O_4_ heterodimer nanocrystals and their applications to a magnetically recyclable catalyst for efficient and selective reduction of nitroarenes and alkenes. Chem. Commun..

[66] Feng Y-S, Ma J-J, Kang Y-M, Xu H-J (2014). PdCu nanoparticles supported on graphene: an efficient and recyclable catalyst for reduction of nitroarenes. Tetrahedron.

